# 1g versus 2 g daily intravenous ceftriaxone in the treatment of community onset pneumonia – a propensity score analysis of data from a Japanese multicenter registry

**DOI:** 10.1186/s12879-019-4552-8

**Published:** 2019-12-26

**Authors:** Shinya Hasegawa, Ryuichi Sada, Makito Yaegashi, Konosuke Morimoto, Takahiro Mori, on behalf of the Adult Pneumonia Study Group-Japan, Masahiko Abe, Takao Wakabayashi, Masahiro Aoshima, Naoto Hosokawa, Norihiro Kaneko, Naoko Katsurada, Kei Nakashima, Yoshihito Otsuka, Eiichiro Sando, Kaori Shibui, Daisuke Suzuki, Kenzo Tanaka, Kentaro Tochitani, Makito Yaegashi, Masayuki Chikamori, Naohisa Hamashige, Masayuki Ishida, Hiroshi Nakaoka, Norichika Aso, Hiroyuki Ito, Kei Matsuki, Yoshiko Tsuchihashi, Koya Ariyoshi, Bhim G. Dhoubhadel, Akitsugu Furumoto, Sugihiro Hamaguchi, Tomoko Ishifuji, Shungo Katoh, Satoshi Kakiuchi, Emi Kitashoji, Takaharu Shimazaki, Motoi Suzuki, Masahiro Takaki, Konosuke Morimoto, Kiwao Watanabe, Lay-Myint Yoshida

**Affiliations:** 1 Department of Infectious Disease, Tokyo Metro Tama Medical Center, 2-8-29 Musashidai, Fuchu, Tokyo, 183-8524 Japan; 2 Department of General Internal Medicine, Kameda Medical Center, 929 Higashi-cho, Kamogawa, Chiba, 296-8602 Japan; 3 Department of General Internal Medicine, Tenri Hospital, 200 Mishima-cho, Tenri, Nara, 632-8552 Japan; 4 Department of Clinical Medicine, Institute of Tropical Medicine, Nagasaki University, 1-14 Bunkyo-cho, Nagasaki, Nagasaki 852-8521 Japan; 5 Department of Health Services Research, Faculty of Medicine, University of Tsukuba, 1-1-1 Tenno-dai, Tsukuba, Ibaraki, 305-8575 Japan; 6 Health Services Research and Development Center, University of Tsukuba, 1-1-1 Tenno-dai, Tsukuba, Ibaraki, 305-8575 Japan; 7 Department of General Internal Medicine, Eastern Chiba Medical Center, 3-6-2 Okayamadai, Togane, Chiba, 283-8686 Japan

**Keywords:** Ceftriaxone, Community-onset pneumonia, Community-acquired pneumonia, Cure rate, Non-inferiority

## Abstract

**Background:**

Community-onset pneumonia (COP) is a combined concept of community acquired pneumonia and the previous classification of healthcare-associated pneumonia. Although ceftriaxone (CRO) is one of the treatment choices for COP, it is unclear whether 1 or 2 g CRO daily has better efficacy. We compared the effectiveness of 1 g with 2 g of CRO for COP treatment. We hypothesized that 1 g CRO would show non-inferiority over 2 g CRO.

**Methods:**

This study was an analysis of prospectively registered data of the patients with COP from four Japanese hospitals (the Adult Pneumonia Study Group-Japan: APSG-J). We included subjects who were initially treated solely with 1 or 2 g of CRO. The propensity score was estimated from the 33 pre-treatment variables, including age, sex, weight, pre-existing comorbidities, prescribed drugs, risk factors for aspiration pneumonia, vital signs, laboratory data, and a finding from chest xrays. The primary endpoint was the cure rate, for which a non-inferiority analysis was performed with a margin of 0.05. In addition, we performed three sensitivity analyses; using data limited to the group in which CRO solely was used until the completion of treatment, using data limited to inpatient cases, and performing a generalized linear mixed-effect logistic regression analysis to assess the primary outcome after adjusting for random hospital effects.

**Results:**

Of the 3817 adult subjects with pneumonia who were registered in the APSG-J study, 290 and 216 were initially treated solely with 1 or 2 g of CRO, respectively. Propensity score matching was used to extract 175 subjects in each group. The cure rate was 94.6 and 93.1% in the 1 and 2 g CRO groups, respectively (risk difference 1.5%; 95% confidence interval − 3.1 to 6.0; *p* = 0.009 for non-inferiority). The results of the sensitivity analyses were consistent with the primary result.

**Conclusions:**

The propensity score-matched analysis of multicenter cohort data from Japan revealed that the cure rate for COP patients treated with 1 g daily CRO was non-inferior to that of patients treated with 2 g daily CRO.

## Background

Community-acquired pneumonia (CAP) is one of the most common infectious diseases and leads to morbidity and mortality worldwide [1, 2]. The Infectious Diseases Society of America (IDSA) guidelines recommend that either respiratory quinolone or beta-lactam plus azithromycin to be used as a first line therapy for CAP; Ceftriaxone (CRO) is one of the recommended regimens among beta-lactam antibiotics [2, 3].

The Sanford Guide for Antimicrobial Therapy recommends that the dose of CRO should be 1–2 g daily to treat pneumonia [4]. However, it is unclear whether 1 or 2 g of CRO daily is better. To date there have been a few studies comparing the effectiveness of 1 and 2 g CRO for CAP. In one study CRO was used for pneumonia in addition to other community acquired infections such as urinary tract infections or cellulitis [5]. Another study compared 1 g daily CRO not only with 2 g daily CRO but also with other agents [6]; with such designs it is not possible to determine the optimal dose of CRO for pneumonia. Previous studies have shown that CRO can cause gallstone formation and a study suggests that more than 2 g or 40 mg/kg daily of CRO is one of the risk factors of gallstone formation (odds ratio: 11.9, 95% confidence interval [CI]: 2.6–54.2) [7, 8]. In addition, optimization of antimicrobial dosing is an essential part of antimicrobial stewardship [9]. Conversely, we need to be very cautious about the effectiveness of antibiotics for the treatment of pneumonia, as the proportion of elderly people in populations is increasing dramatically worldwide, and old age is thought to be an independent risk factor for mortality associated with CAP [10, 11].

Community-onset pneumonia (COP) is a combined concept of CAP and the previous classification of healthcare-associated pneumonia (HCAP). The concept of HCAP was removed in the hospital acquired pneumonia (HAP) / ventilator-associated pneumonia (VAP) guidelines in 2016, as the patients with HCAP frequently present from the community and are initially cared in emergency departments [12].

The aim of our study was to compare the effectiveness of 1 and 2 g daily CRO as treatment for COP, using data from a Japanese multicenter registry. We hypothesized that 1 g CRO would show non-inferiority over 2 g CRO for treatment of patients with COP.

## Methods

### Setting and study population

This propensity score-matching study was a sub-study of the Adult Pneumonia Study Group-Japan (APSG-J) study; this study aimed to compare the effectiveness of 1 g CRO treatment to 2 g of COP treatment for adult patients. The APSG-J study was initiated after obtaining approval by the Institutional Review Boards (IRBs) of all five study hospitals. Written consents from participants were waived by all IRBs because of the study’s observational nature, without any deviation from the current medical practice. The study was conducted on all of the four main islands of Japan from September 2011 through August 2014.

The APSG-J study collected data from adult patients with pneumonia prospectively to elucidate trends in COP and its etiologies in the aging society [13]. Eligible patients were enrolled in the APSG-J study if they fulfilled all of the following criteria: patients 1) ≥ 15 years; 2) exhibited symptoms compatible with pneumonia (e.g., fever, cough, sputum, pleuritic chest pain, or dyspnea); and 3) displayed new pulmonary infiltrates on chest X-ray images (CXR) or computed tomography scans that were consistent with pneumonia. Patients were enrolled from both inpatient and outpatient services. In our study, subjects who were initially treated solely with 1 or 2 g daily CRO were enrolled.

### Assessment of outcomes

The primary outcome was the cure rate, which was defined based on the state on discharge in the patient’s record; this was assessed using the frequency of cured patients in each group. The states in the record included cure, stable condition, exacerbation, death, hospital transfer, and others. Secondary outcomes included in-hospital mortality, the duration of antibiotics, and length of hospital stay between the two groups.

### Data preparation and sample size estimation

All statistical analyses were performed with the R 3.2.3 software for statistical computing (https://www.r-project.org/); the add-on packages “mice” for multiple imputation and “matching” for propensity score matching were used [14, 15]. These analyses were conducted according to the methods used in a previous propensity-score matching study [16]. The primary analysis of the cure rate was conducted using a non-inferiority analysis with a one-sided alpha level of 0.05. The non-inferiority margin was set at an absolute value of 5.0%, based on the Food and Drug Administration non-inferiority clinical trial guidance to determine the margin for pneumonia, and two previous clinical trials [5, 6, 17]. We chose the Farrington and Manning test because propensity-score matching would only give us the same sample size for the two groups, and the Farrington and Manning test requires that the same sample size be enrolled in the two groups. The sample size for the primary outcome was calculated based on the previous randomized control trials, which suggested that the cure rate in the 1 and 2 g CRO groups would be 92 and 87%, respectively [5, 6]. We calculated that a sample of 161 patients per group would give the study 90% power to detect non-inferiority for 1 g CRO treatment.

Apart from the primary outcome of the main analysis, we also used a two-sided alpha level of 0.05, and differences were considered significant if *p*-values were ≤ 0.05. The survival of patients was shown using a Kaplan-Meier survival curve, and adjusted hazard ratios (aHRs) were calculated using multivariable Cox proportional hazards regression analyses. As there were several missing values observed (Additional file 1: Table S1), we used multiple imputation by employing chained equations to complement all missing values for each study variable. Thereby we generated 25 datasets with 20 iterations.

### Propensity score matching

Logistic regression analyses were used to estimate the propensity scores, which were then utilized to predict the efficacy of the use of 1 g over 2 g of CRO. This prediction incorporated 33 pre-treatment covariates, including age, sex, weight, pre-existing comorbidities, if the medical histories were consistent with CAP or not, prescribed drugs prior to admission (specifically prednisolone, anti-acid drug, and sleeping drug), risk factors for aspiration pneumonia, vital signs (respiratory rate, systolic blood pressure, heart rate, and body temperature), laboratory data (hematocrit and blood urea nitrogen, sodium, glucose, and albumin), and a finding from CXRs (pleural effusion). Propensity score matching was conducted for the selected subjects on a pairwise basis after all propensity scores across the imputed datasets had been averaged and logit-transformed. The match caliper was set to 0.2. We used absolute standardized mean differences (ASMDs) for all variables included in the propensity score estimation to assess the match balance; an ASMD of < 0.1 was defined as an appropriate match balance.

### Sensitivity analysis

We performed three sensitivity analyses as follows. All the outcomes were reassessed using data limited to the group in which CRO solely was used until the completion of treatment. We evaluated the primary outcome exclusively for inpatient cases, given that the treatment environment (i.e., inpatient vs. outpatient) might influence mortality. Finally, we included a generalized linear mixed-effect logistic regression analysis to assess the primary outcome after adjusting for random hospital effects, since antibiotic selection preferences might differ between the hospitals.

## Results

### Baseline characteristics of the participants before and after propensity score matching

**Fig. 1:**
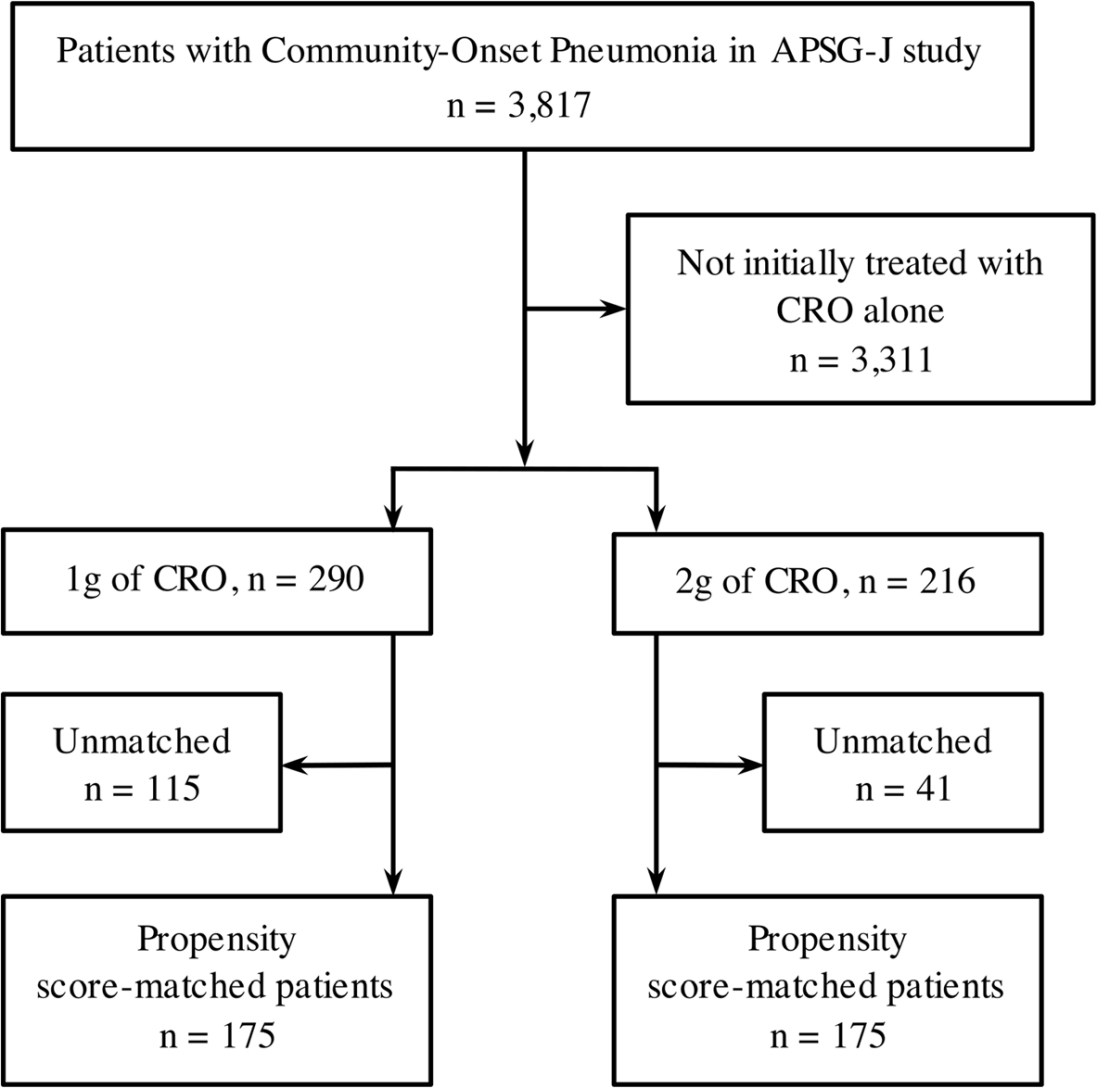
Selection of participants for the study

Of the 3817 adult subjects with pneumonia who were registered in the APSG-J study, 290 and 216 were initially treated solely with 1 or 2 g of CRO, respectively. Propensity score matching was employed to finally extract 175 subjects in each group (Fig. 1, Table 1, Additional file 2: Figure S1).

**Table 1:** Pre-treatment variables for patients initially treated with 1 and 2 g of ceftriaxone included in the propensity score estimation before and after matching

Variables	Before matching			After matching		
	1 g	2 g	ASMD	1 g	2 g	ASMD
	*n* = 290	*n* = 216		*n* = 175	*n* = 175	
Median age (years)	81 [71–87]	78 [67–85]	0.043	79 [70–86]	77 [67–85]	0.054
Sex (Male)	167 (57.6)	146 (67.6)	0.208	117 (66.9)	112 (64.0)	0.060
Median body weight (kg)	50.0 [42.0–59.3]	53.4 [44.0–62.5]	0.176	52.0 [45.0–62.5]	52.6 [44.0–60.8]	0.088
Preexisting comorbidity						
Diabetes mellitus	77 (26.6)	44 (20.4)	0.146	39 (22.3)	38 (21.7)	0.014
Malignancy	54 (18.6)	42 (19.4)	0.021	36 (20.6)	33 (18.9)	0.043
Bronchial asthma	20 (6.9)	27 (12.5)	0.190	18 (10.3)	14 (8.0)	0.079
COPD or bronchiectasis	46 (15.9)	69 (31.9)	0.384	44 (25.1)	45 (25.7)	0.013
Cerebrovascular diseases	59 (20.3)	47 (21.8)	0.035	32 (18.3)	33 (18.9)	0.015
Heart failure	46 (15.9)	47 (21.8)	0.151	32 (18.3)	32 (18.3)	< 0.001
Liver disease	11 (3.8)	14 (6.5)	0.122	9 (5.1)	6 (3.4)	0.085
Kidney disease	31 (10.7)	32 (14.8)	0.124	20 (11.4)	20 (11.4)	< 0.001
Dementia	35 (12.1)	21 (9.7)	0.075	18 (10.3)	18 (10.3)	< 0.001
Prescribed drugs						
Prednisolone	8 (2.8)	13 (6.0)	0.160	8 (4.6)	8 (4.6)	< 0.001
Anti-acid drug	81 (27.9)	79 (36.6)	0.186	55 (31.4)	53 (30.3)	0.025
Sleeping drug	41 (14.4)	30 (13.9)	0.007	25 (14.3)	25 (14.3)	< 0.001
Community- acquired pneumonia	202 (69.7)	160 (74.1)	0.101	132 (75.4)	129 (73.7)	0.039
Risk factors for aspiration pneumonia						
Overt aspiration	35 (12.1)	11 (5.1)	0.251	10 (5.7)	10 (5.7)	< 0.001
Vomiting	3 (1.0)	5 (2.3)	0.100	3 (1.7)	2 (1.1)	0.048
Dysphagia	32 (11.0)	12 (5.6)	0.200	13 (7.4)	11 (6.3)	0.045
Disturbance of consciousness	10 (3.4)	9 (4.2)	0.038	7 (4.0)	7 (4.0)	< 0.001
Neuromuscular diseases	17 (5.9)	5 (2.3)	0.180	3 (1.7)	5 (2.9)	0.077
Tube feeding	59 (20.3)	47 (21.8)	0.035	2 (1.1)	1 (0.6)	0.062
Bedridden status	21 (7.2)	13 (6.0)	0.049	10 (5.7)	10 (5.7)	< 0.001
Vital signs upon arrival at hospital (median)						
RR (breaths/minute)	20 [18–24]	22 [20–26]	0.001	22 [18–26]	22 [18–26]	0.031
SBP (mmHg)	131 [117–149]	131 [118–152]	0.061	133 [118–150]	130 [114–150]	0.043
PR (beats/minute)	94 [84–107]	96 [83–110]	0.093	95 [84–109]	94 [83–110]	0.018
BT (°C)	37.5 [36.8–38.3]	37.5 [36.7–38.1]	0.087	37.5 [36.8–38.1]	37.5 [36.7–38.1]	0.013
Laboratory data (median) and a chest xray finding at admission						
Hct (%)	37.0 [33.1–40.5]	36.9 [33.2–40.7]	0.023	37.1 [33.5–40.8]	36.9 [33.4–41.2]	0.026
BUN (mg/dL)	18.0 [14–24]	17.9 [13.35–26]	0.025	17.0[13.4–25.0]	17.0[13.4–26.0]	0.056
serum Na (mEq/L)	138 [135–140]	138 [136–140]	0.033	139 [135–140]	138 [135–140]	0.027
Glu (mg/dL)	124 [106–153]	129 [109–154]	0.018	125 [109–157]	131 [110–159]	0.038
Alb (mg/dL)	3.4 [3.1–3.8]	3.5 [3.2–3.9]	0.165	3.5 [3.1–3.9]	3.5 [3.1–3.9]	0.033
Pleural effusion on chest xray	18 (6.2)	14 (6.5)	0.011	11 (6.3)	11 (6.3)	< 0.001

Note: Data presented as No. (%) or median [interquartile range]. *ASMDs* absolute standardized mean differences; *COPD* chronic obstructive pulmonary disease; *RR* respiratory rate; *SBP* systolic blood pressure; *PR* pulse rate; *BT* body temperature; *Hct* hematocrit; *BUN* blood urea nitrogen; *Na* sodium; *Glu* glucose; *Alb* albumin

### Primary outcome for patients after propensity score matching

Overall, the cure rate was 94.6% in the 1 g CRO group and 93.1% in the 2 g CRO group (risk difference 1.5%; 95% CI − 6.6 to 3.6; *p* = 0.009 for non-inferiority; *p* = 0.572 for superiority) (Table 2).

**Table 2:** Main analysis to compare primary and secondary outcomes for the patients who received 1 and 2 g of ceftriaxone for the treatment of community-onset pneumonia

	1 g (*n* = 175)	2 g (*n* = 175)	absolute difference	*p* value
Primary Outcome				
Cure rate (%)	94.6	93.1	1.5 (−3.1, 6.0)	0.009 for non-inferiority, 0.572 for superiority
Secondary Outcomes				
Length of hospital stay (days)	17	26	–9 (−4, −14)	< 0.001
Duration of antibiotic treatment (days)	8	10	–2 (−1, −3)	0.002
In-hospital mortality (%)	4.7	4.0	0.7 (−3.6, 5.0)	0.740

### Secondary outcomes for patients after propensity score matching

**Fig. 2:**
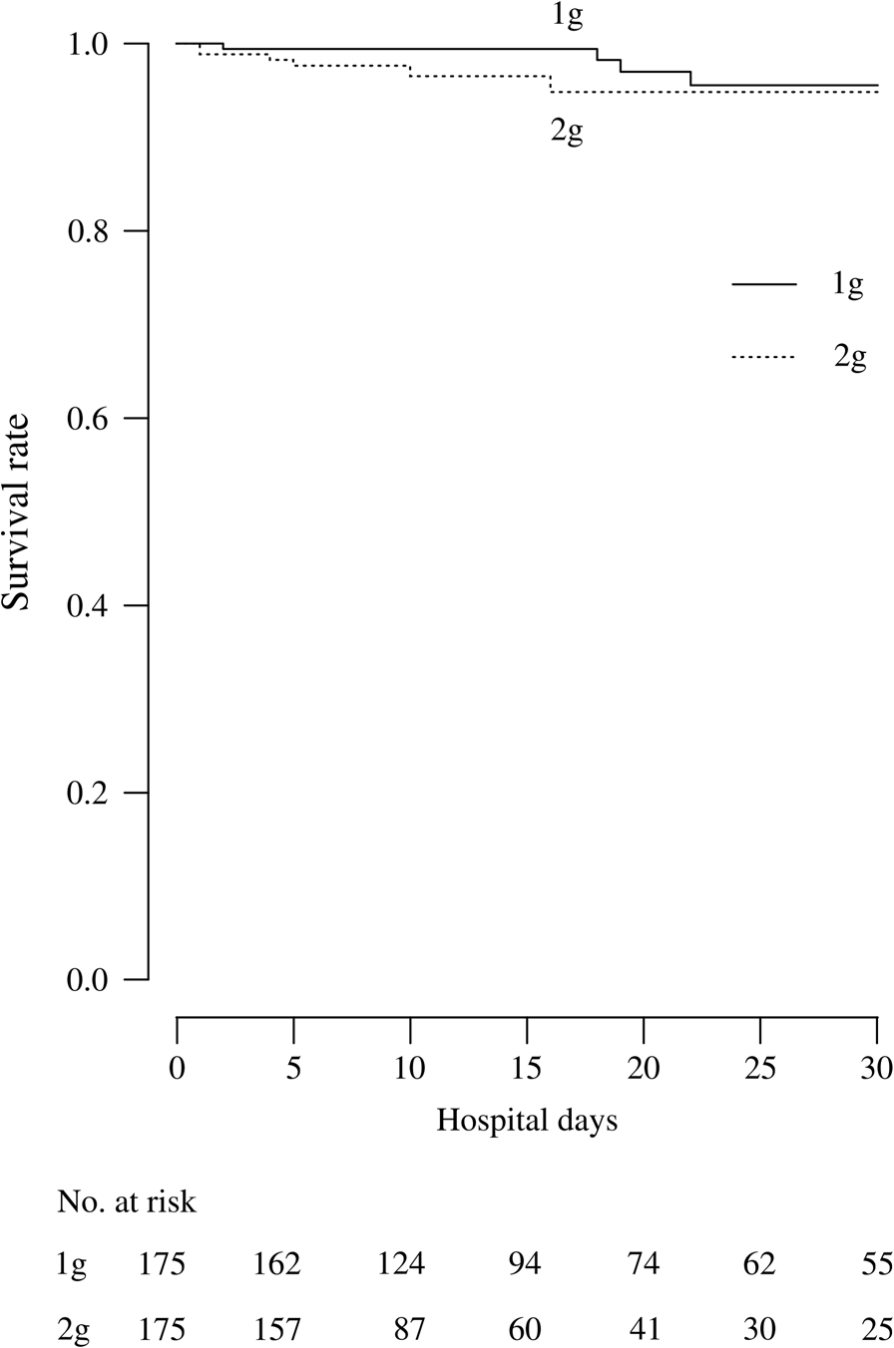
Survival curves for propensity score-matched subjects with community-onset pneumonia initially treated with 1 and 2 g of ceftriaxone

When considering all of the propensity score-matched subjects, the length of hospital stay in the 1 g CRO group (17 days [95% CI: 14–21 days]) was significantly shorter than that in the 2 g CRO group (26 days [95% CI: 22–30 days]; *p* < 0.001). Duration of antibiotic treatment in the 1 g CRO group was also significantly shorter (8 days [95% CI: 8 − 9 days]) than that in the 2 g CRO group (10 days [95% CI: 9–10 days]; *p* = 0.002). The in-hospital mortality rate did not significantly differ between the 1 (4.7% [95% CI: 1.5–8.0%]) and 2 g CRO groups (4.0% [95% CI: 1.1–6.9%]; *p* = 0.740) (Table 2). Survival analysis of the propensity score-matched subjects revealed a similar survival time in the two groups. Specifically, the aHR for mortality in the 1 g CRO group vs. the 2 g CRO group was 1.58 (95% CI: 0.56–4.43) (*p* = 0.385) (Fig. 2).

### Results of the sensitivity analyses

For the patients in which treatment was solely CRO for the duration of treatment, we could not ascertain an appropriate match using the same variables for the main analysis; thus, we decreased the number of variables used, limiting the sample size to 94 subjects in each group. The cure rate was 88.9% (95% CI: 82.3–95.4%) in the 1 g CRO group and 91.5% (95% CI: 85.7–97.2%) in the 2 g CRO group (*p* = 0.549 for superiority). The difference in the length of hospital stay was significantly shorter in the 1 g CRO group (18 days [95% CI: 14 − 22 days]) than that in the 2 g CRO group (26 days [95% CI: 21–32 days]; *p* = 0.007). The duration of antibiotics treatment and the in-hospital mortality rate did not significantly differ between the two groups (Table 3). The analysis using only inpatient cases produced similar results (1 g CRO group: 95.4% [95% CI: 91.8–99.0%] vs. 2 g CRO group: 90.7% [95% CI: 85.8–95.6%]; *p* = 0.127 for superiority). Finally, the analysis using the random hospital effects as a sensitivity measure also supported the above finding (odds ratio, 0.77 [95% CI: 0.32–1.90], *p* = 0.576).

**Table 3:** Sensitivity analysis to compare primary and secondary outcomes for the patients who received 1 and 2 g of ceftriaxone solely for the treatment of community-onset pneumonia

	1 g (*n* = 94)	2 g (*n* = 94)	absolute difference	*p* value
Primary Outcome				
Cure rate (%)	88.9	91.5	−2.6 (−6.0, 11.3)	0.549 for superiority
Secondary Outcomes				
Length of hospital stay (days)	18	26	−9 (−2, −15)	0.007
Duration of antibiotic treatment (days)	7	8	0 (−1, 1)	0.589
In-hospital mortality (%)	7.9	3.2	4.7 (−2.0, 11.3)	0.168

## Discussion

In this study, the cure rate for COP patients treated with 1 g daily CRO was non-inferior to those treated with 2 g daily CRO. The aim of this study was to show that the smaller antibiotic dose was non-inferior to the higher dose. Although the optimization of antibiotics dose is one of the major aims in the era of antimicrobial stewardship [9] in order to make resistant pathogens less likely to occur, many clinicians and researchers alike may consider the difference between treatment with 1 g and that with 2 g CRO as subtle. To our knowledge, high-quality evidence regarding the difference in efficacy between 1 and 2 g of CRO is lacking. Although a few studies recently evaluated the effectiveness of 1 and 2 g CRO and some other agents for CAP [17, 18], the numbers of patients enrolled were small, leading to a lack of power [5, 6]. Our study will further enhance the results of the previous clinical trials, given that our study was performed with adequate power.

We were aware that determining the non-inferiority margin should be prudent and cautious, and it is important that the margin is set appropriately based on the findings from previous studies [19, 20]. We set 5% as the non-inferiority margin for the cure rate based on the findings from previous clinical trials [5, 6]. There is a trade-off relationship between the non-inferiority margin and the potential benefits, which in our study included promoting the antimicrobial stewardship and potential fewer side effects (e.g., gallstone formation). We believe that the 5% non-inferiority margin was a reasonable value from a clinical standpoint.

The length of hospital stay and duration of antibiotics were significantly shorter in the 1 g CRO group based on the main analysis, and the sub analysis showed that the length of hospital stay was also significantly shorter in the 1 g CRO group, in which the treatment was completed exclusively using 1 g CRO. As these results were unexpected, we conducted a generalized linear mixed-effect logistic regression model analysis, and confirmed that the length of the hospital stay and duration of antibiotics were not influenced by random hospital effects. These results might be explained by the fact that a lower dose of antibiotics might lead to fewer side effects, because a variety of adverse events related to CRO were reported [21]. We are, however, uncertain whether all of those adverse events, with the exception of gallstones, were dose-dependent [8, 22]. In this study, we did not have information regarding the side effects caused by antibiotic treatment reported in the various hospitals. Another possible explanation for our findings is that patients’ socioeconomic status (SES) in the 1 g CRO group could have been higher compared to those in the 2 g CRO group. The higher the SES and the fewer problems encountered by the family in preparing a well-ordered environment or nursing home for the patient, the greater the chances might be that the patient would be discharged earlier. These factors suggest that our results should be interpreted prudently.

This study was subject to several limitations. First, this study was designed as non-inferiority trial, and the margin was set retrospectively. However, we discussed the appropriateness of the non-inferiority margin carefully. Second, this was an observational study, and the information was not collected regarding the factors related to SES. Third, we were unable to make comparisons between the two groups based on the sputum cultures. Our results may be associated with differences in bacterial etiology, so their interpretation requires some caution. However, we could not include culture results as pre-treatment variables, as these results were obtained after treatment was initiated, i.e., we usually prescribed antibiotics without any significant sputum culture results since approximately 3 days are required to identify pathogens. The frequency of pneumonia related bacteria in the APSG-J study population was previously reported [13]. Fourth, the overall in-hospital mortality rate in our study was lower (4.3%) than that of a COP mortality (11.5%) in the APSG-J study [13]. This might reflect the situation, in which our participants might be healthier than typical COP patients.

Our study had several strengths despite these limitations. To our knowledge, this was the first study to consider the body weight of the patient to investigate the optimal dose of CRO. The mechanism in which CRO is distributed throughout the body depends on the patient's body weight, so we would not be able to investigate the effectiveness of CRO without also taking body weight into account. Second, we used prospectively collected multicenter registry data; multiple imputation and propensity score matching were conducted to increase the robustness of the analysis. Additionally, many covariates were analyzed to increase the consistency of the results. We chose a variety of covariates not only based on major criteria such as the CURB-65 or Pneumonia Severity Index [23, 24], but we also included factors associated with pneumonia mortality [25–27]. Finally, our study had sufficient power as discussed above, while one of the major criticisms of the previous studies was the insufficient power. This study, therefore, provided additional meaningful insights regarding the optimal dose of CRO for the treatment of COP.

## Conclusions

In this propensity score-matched analysis of multicenter cohort data, the cure rate of COP patients treated with 1 g daily CRO was non-inferior to those treated with 2 g daily CRO. Our study offers useful insights regarding the optimal dose of CRO for patients with COP. Further studies, for example randomized control studies with adequate power are needed to strengthen the evidence regarding this treatment alternative.

## Supplementary information

**Additional file 1: Table S1.** The number of missing values of pretreatment variables in patients with aspiration-associated pneumonia.

**Additional file 2: Figure S1.** The distributions of the propensity scores before and after matching.

## Data Availability

The datasets used and/or analyzed during the current study are not publicly available due to other ongoing research projects using the datasets, but could be available from the corresponding author on reasonable request.

## Abbreviations

aHRs: Adjusted hazard ratios
APSG-J: Adult Pneumonia Study Group-Japan
ASMDs: Absolute standardized mean differences
CAP: Community-acquired pneumonia
COP: Community-onset pneumonia
CRO: Ceftriaxone
CXR: Chest xray
HAP: Hospital-acquired pneumonia
HCAP: Healthcare-associated pneumonia
IDSA: Infectious Diseases Society of America
IRBs: Institutional Review Boards
SES: Socioeconomic status
VAP: Ventilator-associated pneumonia
