# Supplementary material for: 1g versus 2 g daily intravenous ceftriaxone in the treatment of community onset pneumonia – a propensity score analysis of data from a Japanese multicenter registry
Source: BMC Infect Dis. 2019 Dec 26;19:1079. doi: 10.1186/s12879-019-4552-8 (PMC6933656; doi:10.1186/s12879-019-4552-8)
Supplement: Supplementary file 1 — Additional file 1: Table S1. The number of missing values of pretreatment variables in patients with aspiration-associated pneumonia. [file 12879_2019_4552_MOESM1_ESM.docx]

**Additional file 1: Table S1.**

**The number of the missing values of the study variables**

| **Variables** | **The number of the missing values** | |
| --- | --- | --- |
|  | 1 g | 2 g |
|  | n = 290 | n = 216 |
| **Age** | 0 | 0 |
| **Sex** | 0 | 0 |
| **Body weight** | 33 | 35 |
| **Preexisting comorbidity** |  |  |
| Diabetes mellitus | 0 | 0 |
| Malignancy | 0 | 0 |
| Bronchial asthma | 0 | 0 |
| COPD or bronchiectasis | 0 | 0 |
| Cerebrovascular diseases | 0 | 0 |
| Heart failure | 0 | 0 |
| Liver disease | 0 | 0 |
| Kidney disease | 0 | 0 |
| Dementia | 0 | 0 |
| **Prescribed drugs** |  |  |
| Prednisolone | 0 | 0 |
| Anti-acid drug | 0 | 0 |
| Sleeping drug | 0 | 0 |
| **Community-acquired pneumonia** | 1 | 0 |
| **Risk factors for aspiration pneumonia** |  |  |
| Overt aspiration | 0 | 0 |
| Vomiting | 0 | 0 |
| Dysphagia | 0 | 0 |
| Disturbance of consciousness | 0 | 0 |
| Neuromuscular diseases | 0 | 0 |
| Tube feeding | 0 | 0 |
| Bedridden status | 0 | 0 |
| **Vital signs upon arrival at hospital** |  |  |
| RR | 14 | 39 |
| SBP | 1 | 4 |
| PR | 1 | 3 |
| BT | 1 | 1 |
| **Laboratory data and chest xray findings at admission** | | |
| Hct | 1 | 0 |
| BUN | 1 | 1 |
| serum Na | 2 | 2 |
| Glu | 26 | 22 |
| Alb | 11 | 7 |
| Pleural effusion on chest xray | 0 | 0 |

Note: COPD: chronic obstructive pulmonary disease; RR: respiratory rate; SBP: systolic blood pressure; PR: pulse rate; BT: body temperature; Hct: hematocrit; BUN: blood urea nitrogen; Na: sodium; Glu: glucose; Alb: albumin
