# Supplementary material for: 1g versus 2 g daily intravenous ceftriaxone in the treatment of community onset pneumonia – a propensity score analysis of data from a Japanese multicenter registry
Source: BMC Infect Dis. 2019 Dec 26;19:1079. doi: 10.1186/s12879-019-4552-8 (PMC6933656; doi:10.1186/s12879-019-4552-8)
Supplement: Supplementary file 2 — Additional file 2: Figure S1. The distributions of the propensity scores before and after matching. [file 12879_2019_4552_MOESM2_ESM.docx]

**Additional file 2: Figure S1.**

**The distributions of the propensity scores before and after matching**


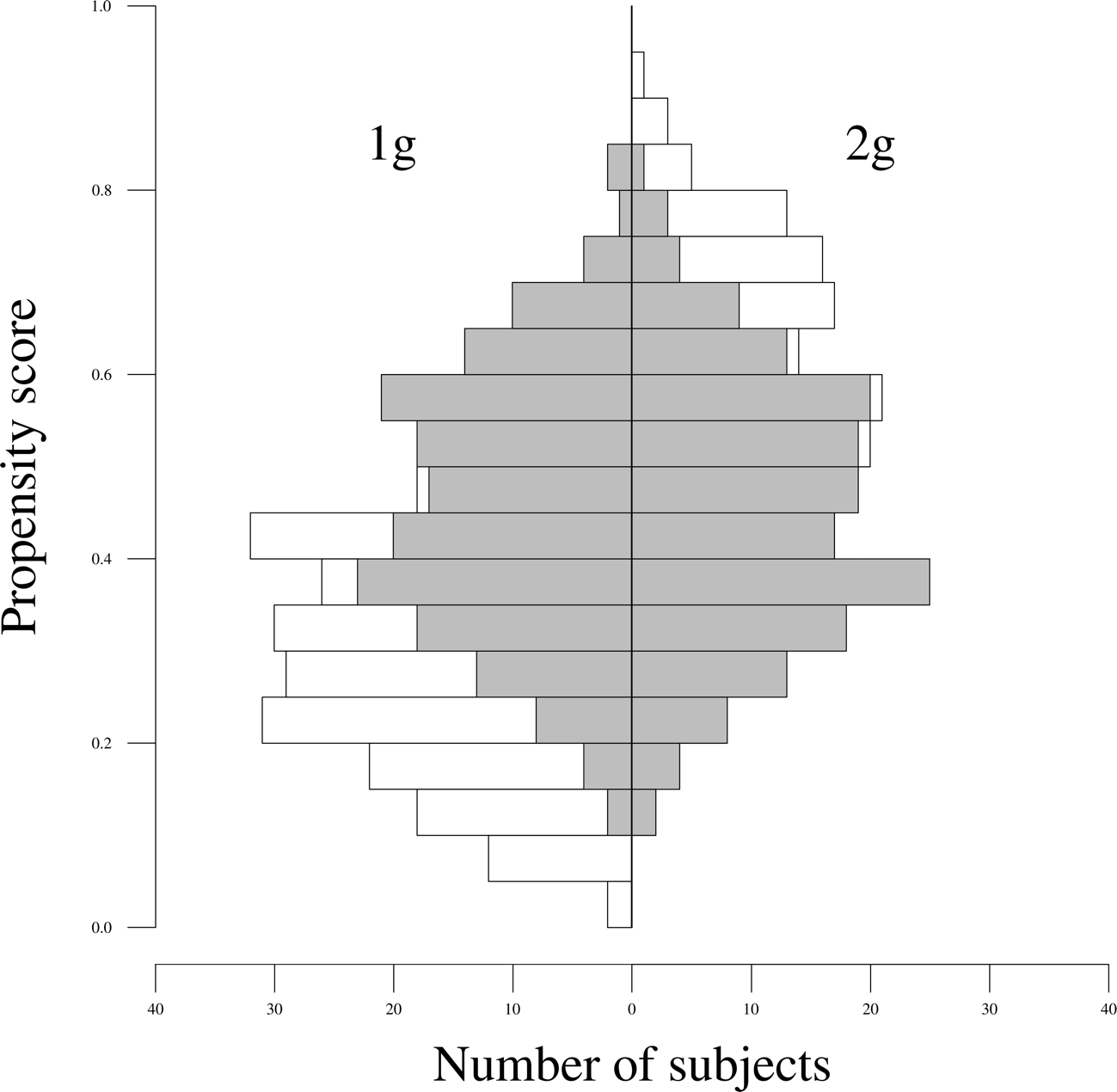


Note: The histogram is based on 5% steps in the propensity score. White bars represent the data before matching and the gray bars represent the data after matching.
